# Impact of the Smoothened Inhibitor, IPI-926, on Smoothened Ciliary Localization and Hedgehog Pathway Activity

**DOI:** 10.1371/journal.pone.0090534

**Published:** 2014-03-07

**Authors:** Marisa O. Peluso, Veronica T. Campbell, Joseph A. Harari, Thomas T. Tibbitts, Jennifer L. Proctor, Nigel Whitebread, James M. Conley, Kerry F. White, Jeffery L. Kutok, Margaret A. Read, Karen McGovern, Kerrie L. Faia

**Affiliations:** Infinity Pharmaceuticals, Inc., Cambridge, Massachusetts, United States of America; Institute of Hepatology, Foundation for Liver Research, United Kingdom

## Abstract

A requisite step for canonical Hedgehog (Hh) pathway activation by Sonic Hedgehog (Shh) ligand is accumulation of Smoothened (Smo) to the primary cilium (PC). Activation of the Hh pathway has been implicated in a broad range of cancers, and several Smo antagonists are being assessed clinically, one of which is approved for the treatment of advanced basal cell carcinoma. Recent reports demonstrate that various Smo antagonists differentially impact Smo localization to the PC while still exerting inhibitory activity. In contrast to other synthetic small molecule Smo antagonists, the natural product cyclopamine binds to and promotes ciliary accumulation of Smo and “primes” cells for Hh pathway hyper-responsiveness after compound withdrawal. We compared the properties of IPI-926, a semi-synthetic cyclopamine analog, to cyclopamine with regard to potency, ciliary Smo accumulation, and Hh pathway activity after compound withdrawal. Like cyclopamine, IPI-926 promoted accumulation of Smo to the PC. However, in contrast to cyclopamine, IPI-926 treatment did not prime cells for hyper-responsiveness to Shh stimulation after compound withdrawal, but instead demonstrated continuous inhibition of signaling. By comparing the levels of drug-induced ciliary Smo accumulation with the degree of Hh pathway activity after compound withdrawal, we propose that a critical threshold of ciliary Smo is necessary for “priming” activity to occur. This “priming” appears achievable with cyclopamine, but not IPI-926, and is cell-line dependent. Additionally, IPI-926 activity was evaluated in a murine tumor xenograft model and a pharmacokinetic/pharmacodynamic relationship was examined to assess for in vivo evidence of Hh pathway hyper-responsiveness. Plasma concentrations of IPI-926 correlated with the degree and duration of Hh pathway suppression, and pathway activity did not exceed baseline levels out to 96 hours post dose. The overall findings suggest that IPI-926 possesses unique biophysical and pharmacological properties that result in Hh pathway inhibition in a manner that differentiates it from cyclopamine.

## Introduction

The hedgehog (Hh) pathway plays a role both in embryonic development and adult stem cell function. It is also involved in cancer, exemplified by basal cell carcinoma and medulloblastoma where genetic mutations lead to constitutive activation of the pathway [1]. The Hh signaling pathway is complex and involves two transmembrane proteins, Patched (Ptc) and Smoothened (Smo), and is regulated by the absence or presence of Hh ligands. For most adult cells, Hh ligand is absent, and Ptc functions to repress Smo activity, keeping the pathway inactive. Upon binding of Hh ligand to Ptc, Smo inhibition is relieved. Derepression of Smo triggers a signal transduction cascade that activates Gli transcription factors resulting in expression of target genes that regulate cellular survival, differentiation, migration, and proliferation [2].

The precise biochemical events that are involved in Hh ligand-mediated activation of mammalian cells are still being elucidated. However, the primary cilium (PC), a specialized form of non-motile cilia, is essential for transduction of the Hh signal in mammalian cells [3]. The process of Hh signaling is regulated not just at the level of protein activity, but also by localization of the key signaling proteins to the PC. In the absence of Hh ligand, Ptc localizes to the PC and inhibits Smo by preventing its accumulation within the PC. When Ptc binds Hh ligand, it leaves the PC and is internalized through endosomes, leading to accumulation of Smo in the PC and activation of signaling [4]. Studies with yellow fluorescent protein (YFP)-tagged Smo have demonstrated that the movement of Smo to the PC is through lateral transport from the plasma membrane, which differs from the movement of most ciliary proteins in mammalian cilia [5]. Additional work led to the proposal of a two-step activation process where movement of Smo to the PC is not sufficient for pathway activation and an additional step is necessary for Smo to activate the pathway [6]. These studies showed that overexpression of Smo can result in ciliary localization without activating signaling [6]. Hh ligands, SMO agonists as well as the Smo antagonist, cyclopamine promote Smo accumulation within the PC [7]. Smo localization to the PC in murine NIH-3T3 cells is blocked by some small molecule inhibitors of Smo, such as SANT1, SANT2 and GDC-0449 [6], [8]. While Smo was still present in the PC following treatment with the natural product Smo inhibitor cyclopamine, Hh signaling was not activated. These reports provide evidence that localizing Smo to the PC is necessary, but may not be sufficient for activation [6], [8].

The ability of Smo to localize to the PC even in the presence of cyclopamine led to the concern that, in the setting of administration of Smo inhibitors in the clinic, cells could become hypersensitive to renewed signaling as the inhibitor concentration declines. In other words, if the pathway is inhibited in cells by a Smo antagonist that retains Smo in the PC, would these cells then be “primed” for high levels of activation when re-exposed to ligand? Studies were performed with different Smo antagonists in the NIH-3T3 cell line that showed robust signaling when exposed to Hh ligand [8]. As expected, all inhibitors blocked Hh pathway activation in NIH-3T3 cells as measured with a Gli-luciferase reporter. However, if inhibitors were withdrawn and then cells were re-activated with Hh ligand, the cells pre-exposed to cyclopamine expressed higher levels of Gli activity compared to levels in untreated cells. This finding suggests that differences among Smo inhibitor compounds can lead to different degrees of pathway reactivation, at least in the NIH-3T3 cells.

IPI-926 is a novel semi-synthetic derivative of the natural product cyclopamine that directly binds to and blocks the activity of Smo [9]. IPI-926 is more potent and selective than cyclopamine and has improved chemical and metabolic stability, solubility and oral bioavailability [9]. IPI-926 is highly effective at inhibiting the Hh pathway in murine and human cells as measured in multiple assays. We examined whether IPI-926 leads to Smo localization to the PC and whether this accumulation “primes” cells to hyper-respond to Hh ligand stimulation after compound withdrawal. We report that like cyclopamine, IPI-926 localizes Smo to the PC and this localization does not lead to pathway activation in the absence of Hh ligand stimulation. In stark contrast to cyclopamine, Smo localization to the PC by IPI-926 does not lead to priming of the cells for hyper-activation when re-exposed to Hh ligand after compound withdrawal. In addition, we report that the priming activity observed with cyclopamine is cell-line specific. Our data suggest that the priming effect that can lead to hyper-activation in NIH-3T3 cells may be due to the cells reaching a threshold level of Smo in the PC. Therefore, despite differences in ciliary Smo localization properties, IPI-926 is similar to other synthetic Smo inhibitors in that it can block Hh pathway activation and does not lead to priming for hyper-activation.

## Results

### IPI-926 is a potent and selective Smo antagonist

**Figure 1:**
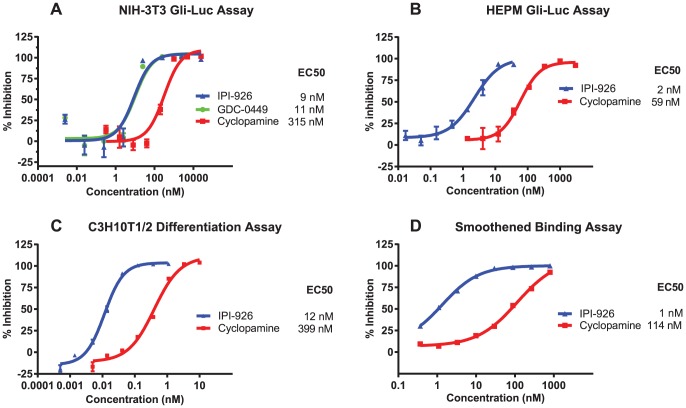
IPI-926 is significantly more potent than cyclopamine in cell based assays. IPI-926 inhibits Shh-induced Gli-Luciferase activity with an EC_50_ value of 9 nM in NIH-3T3 cells (A) and 2 nM in HEPM cells (B) compared to cyclopamine which had an EC_50_ value of 315 nM in NIH-3T3 cells (A) and 59 nM in HEPM cells (B) (N = 3). (C) IPI-926 inhibits alkaline phosphatase production resulting from Shh-induced C3H10T1/2 cell differentiation with an EC_50_ value of 12 nM compared to 399 nM for cyclopamine (N = 2). (D) IPI-926 inhibits the binding of BODIPY-labeled cyclopamine to C3H10T1/2 cells overexpressing wild-type human Smo with an EC_50_ value of 1 nM compared to an EC_50_ value of 114 nM for unlabeled cyclopamine.

To compare the potency of IPI-926 relative to cyclopamine, various cell-based assays were employed. IPI-926 was more potent than cyclopamine (∼30-fold) at inhibiting Gli-luciferase reporter activity in both murine (NIH-3T3, Figure 1A) and human (HEPM, Figure 1B) cell lines containing endogenous Smo. IPI-926 was also more potent than cyclopamine in the C3H10T1/2 differentiation assay (Figure 1C), which is a functional assessment of Hh pathway activity as measured by the production of alkaline phosphatase, a marker of osteoblast differentiation in response to Shh stimulation [10], [11]. Additionally, IPI-926 displayed greater binding affinity (∼80-fold) for expressed human Smo in a BODIPY-cyclopamine competition assay (Figure 1D) [12]. These results demonstrate that IPI-926 is a potent Smo antagonist against both murine and human Smo isoforms. Since IPI-926 is more potent than cyclopamine at inhibiting Smo, the concentrations of IPI-926 (500 nM) and cyclopamine (5 µM) that were used in subsequent assays were selected to achieve maximal pathway inhibition for each compound. The inhibitory activity of GDC-0449 (vismodegib), a synthetic small molecule Smo inhibitor that has been approved by the U.S. Food and Drug Administration (FDA) for treatment of advanced basal cell carcinoma and marketed as Erivedge, is similar to that of IPI-926 in all assays (Figure 1A and data not shown). Therefore, a concentration of 500 nM GDC-0449 was selected for subsequent experiments.

### Ciliary Smo localization upon treatment with Smo antagonists

**Figure 2:**
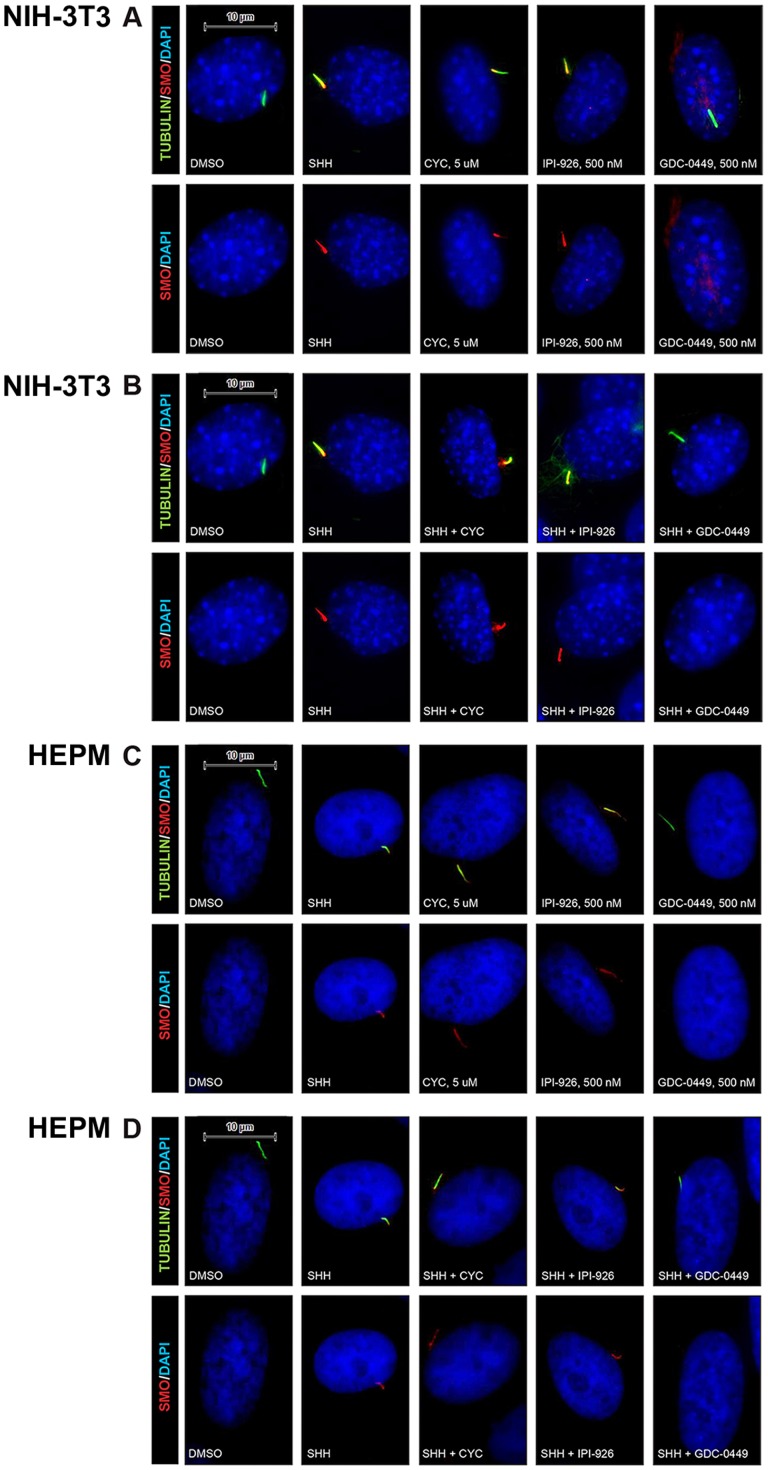
Smo antagonists differentially localize endogenous Smo protein (red) to the PC (green). In both NIH-3T3 (A) and HEPM cells (C), 24 h treatment with either cyclopamine (CYC, 5 µM) or IPI-926 (500 nM) in the absence of Shh, results in Smo accumulation to the PC while treatment with GDC-0449 (500 nM) does not. Shh treatment results in Smo accumulation to the PC in both NIH-3T3 cells (B) and HEPM cells (D), which can be blocked by GDC-0449 but not with cyclopamine or IPI-926.

While multiple pathway agonists (SAG, purmorphaine, oxysterols, Shh ligand) induce Smo localization to the PC [4], [6], [13], various Hh pathway antagonists have also been shown to induce Smo localization to the PC [14]. Cyclopamine is capable of localizing Smo to the PC while retaining inhibitory function [6], [13], [14]. Other classes of Smo antagonists such as GDC-0449 or SANT-1 do not induce Smo localization to the PC, suggesting differential modes of inhibition among various classes of Smo antagonists [6], [14]. The impact of IPI-926 on Smo localization, in the absence and presence of Shh ligand, was evaluated in both murine (NIH-3T3) and human (HEPM) Hh signaling-competent cell lines. Like cyclopamine, IPI-926, at concentrations that abrogate Hh signaling, induced Smo localization to the PC in the absence or presence of Shh ligand in both the NIH-3T3 and HEPM cells (Figure 2A–D). In contrast, this effect on Smo localization in the PC was not seen with GDC-0449 (Figure 2A–D). These data support different modes of inhibition when comparing the natural product cyclopamine analogs vs. synthetic small molecules, such as GDC-0449.

### IPI-926 pre-treatment does not result in hyper-responsiveness to Shh stimulation

**Figure 3:**
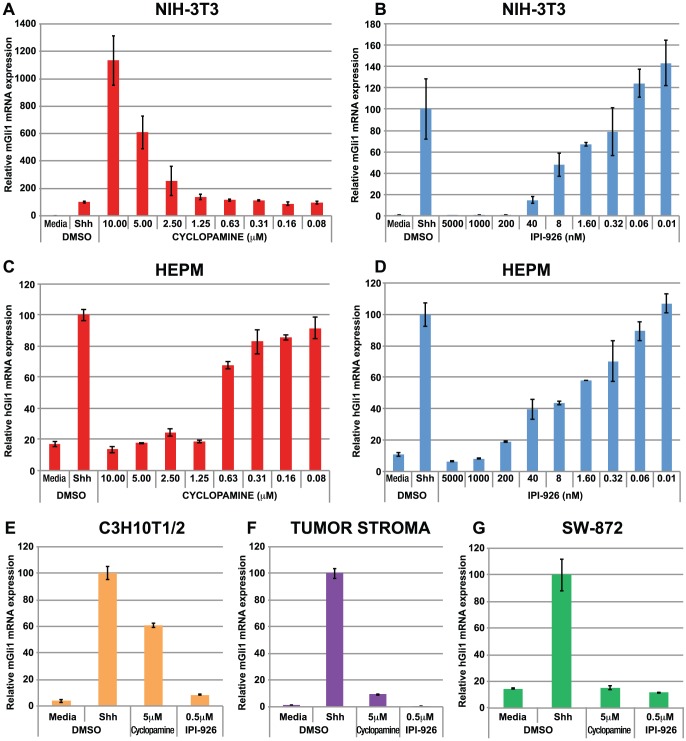
Treatment of NIH-3T3 cells with cyclopamine but not IPI-926 confers hyper-responsiveness to Shh stimulation. Cells were pre-treated with either cyclopamine or IPI-926 for 24 h followed by extensive washing and subsequent treatment with either media alone or media containing 100 nM rShh. Gli1 mRNA levels were assessed by qRT-PCR 21 h post stimulation. Hyper-responsiveness to rShh stimulation is observed in NIH-3T3 cells treated with high doses of cyclopamine (A) but not IPI-926 (B). Note different Y-axis scales between the 2 graphs. Identical experiments carried out in HEPM cells treated with either cyclopamine (C) or IPI-926 (D) does not result in Shh hyper-responsiveness. No Shh hyper-responsiveness was observed in C3H10T1/2 cells (E), pancreatic xenograft tumor stroma (F) or SW-872 cells (G) when pre-treated with 5 µM cyclopamine or 500 nM IPI-926.

A recent report has revealed that in addition to inducing Smo accumulation in the PC, after compound withdrawal, cyclopamine pre-treated cells are more “primed” or hyper-responsive to Hh pathway reactivation [8]. To confirm this finding with cyclopamine, and to investigate whether the cyclopamine analog IPI-926 is also associated with this phenomenon, NIH-3T3 cells were pre-treated with increasing concentrations of cyclopamine or IPI-926 for 24 h, followed by compound withdrawal and extensive rinsing of the cells. The cells were then challenged with Shh ligand and the extent of Hh pathway activation was determined by quantifying Gli1 mRNA levels. As previously described by Wang et al. 2012 [8], pre-treatment of NIH-3T3 cells with high concentrations of cyclopamine resulted in hyper-responsiveness to Shh stimulation after compound removal (Figure 3A). In contrast, IPI-926 pre-treatment did not render NIH-3T3 cells hyper-responsive to Hh pathway stimulation (Figure 3B). To determine if this phenomenon of hyper-responsiveness to Hh pathway stimulation following cyclopamine removal was specific to NIH-3T3 cells, both cyclopamine and IPI-926 were evaluated in human HEPM cells (Figure 3C & D), and three additional Hh signaling-competent cell lines of both human (SW-872) and murine origin (C3H10T1/2 and pancreatic xenograft tumor stroma) (Figure 3E–G). Neither cyclopamine nor IPI-926 pre-treatment resulted in hyper-responsiveness to Shh stimulation after compound removal in any of the additional cell lines tested (Figures 3C–G). These data indicate that cyclopamine mediated “priming” is not a universal phenomenon across all Hh-competent cell lines as it was only observed in the murine NIH-3T3 cell line. Additionally, while IPI-926 and cyclopamine are in the same structural class of compounds, and are both capable of accumulating Smo to the PC, they possess distinct properties in that priming activity is only observed with high doses of cyclopamine, but not IPI-926 in the NIH-3T3 cells. Nonetheless, these results warranted further investigation into the mechanism of hyper-responsiveness with cyclopamine pre-treatment.

### IPI-926 and cyclopamine differ in their ability to induce Smo accumulation in the PC

**Figure 4:**
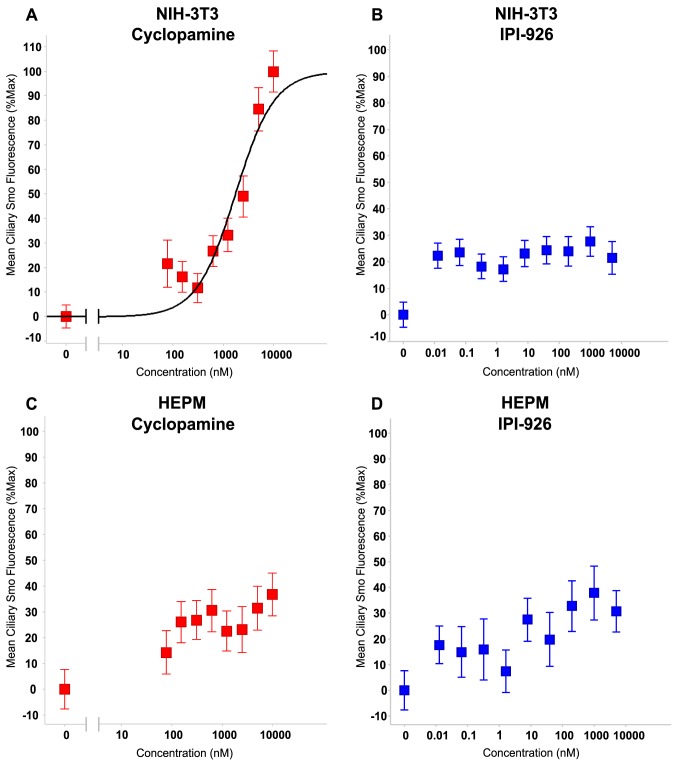
Treatment of NIH-3T3 cells with cyclopamine but not IPI-926 results in increased ciliary Smo accumulation. In the absence of Shh, NIH-3T3 cells were treated for 24 h with either cyclopamine (A) or IPI-926 (B) and the mean fluorescence intensity of ciliary Smo accumulation was calculated. A positive correlation was observed between increasing doses of cyclopamine and mean ciliary Smo fluorescence intensity. Dose responsive Smo accumulation is not observed with IPI-926. Ten µM cyclopamine yielded the maximal Smo fluorescence intensity (63%) and all other data points were normalized to this max value. Dose responsive Smo accumulation is not observed in HEPM cells treated for 24 h with either cyclopamine (C) or IPI-926 (D).

We hypothesized that variances in compound-induced ciliary Smo accumulation may account for the observed dissimilarities in priming activity between IPI-926 and cyclopamine in NIH-3T3 cells. It has been shown that treating NIH-3T3 cells with increasing concentrations of cyclopamine correlates with enhanced ciliary Smo accumulation and inhibition of Gli-reporter activity, supportive of a model whereby cyclopamine induces accumulation of an inactive form of Smo to the PC [6]. To measure the Smo localization to the PC by cyclopamine, and compare to that induced by IPI-926, NIH-3T3 cells were treated with either cyclopamine or IPI-926 at the same concentrations utilized in the priming assays (Figure 3A & B), and mean ciliary Smo fluorescence intensity was quantitated. A concentration-dependent increase in ciliary Smo fluorescence intensity was observed in cyclopamine-treated NIH-3T3 cells (Figure 4A). In contrast, IPI-926 treatment did not result in accumulation of Smo to the PC in a dose-dependent manner (Figure 4B). These data suggest a significant difference in the physical properties of cyclopamine and IPI-926 that may account for the observed differences in compound-induced enrichment of Smo to the PC. To explore the impact of ciliary Smo accumulation with compound treatment in the HEPM cell line, Smo fluorescence intensity was quantitated upon treatment with increasing doses of either cyclopamine or IPI-926. Cyclopamine did not dose-dependently increase Smo accumulation to the PC in HEPM cells (Figure 4C) to the same extent as observed in the NIH-3T3 cells. Consistent with the results obtained in NIH-3T3 cells, IPI-926 treatment did not lead to significant ciliary Smo accumulation in HEPM cells (Figure 4D). Collectively, these data suggest that a certain threshold of Smo accumulation is necessary for hyperactive signaling to occur after compound removal and after exogenous Shh stimulation. Only in the NIH-3T3 cell line were high concentrations of cyclopamine (2.5 to 10 µM) able to induce the critical threshold of ciliary Smo accumulation required for priming activity to occur. Interestingly, these concentrations are well above the EC_50_ value of cyclopamine for inhibition of Gli-reporter activity (Figure 1A).

### IPI-926 does not induce hyperactive signaling in MiaPaCa xenograft model

**Figure 5:**
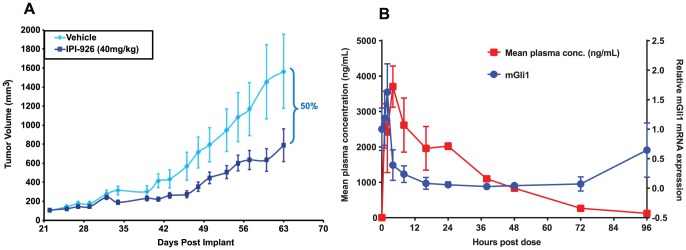
Hyper-responsiveness to paracrine Hh signaling was not observed in IPI-926 treated pancreatic xenografts. (A) In the MiaPaCa pancreatic xenograft model, 50% tumor growth inhibition was observed with daily oral administration of IPI-926 at 40 mg/kg (N = 12 mice per group). (B) Plasma levels of IPI-926 correlate with the degree and duration of stromal mGli1 inhibition after a single dose of IPI-926 at 40 mg/kg. * indicates a p-value <0.003. Modulation of tumor-derived hGli1 was not observed (data not shown).

The MiaPaCa pancreatic xenograft model was used to explore further the effect of IPI-926 treatment on Hh pathway hyperactivity in vivo. This system models paracrine Hh signaling whereby the MiaPaCa tumor cells secrete Hh ligand to surrounding stromal cells leading to enhanced Gli1 transcription [15]. Mice bearing established MiaPaCa tumors were administered 40 mg/kg IPI-926 daily and monitored for tumor growth inhibition. IPI-926 inhibited tumor growth by 50% compared to vehicle treated controls (Figure 5A). A pharmacokinetic/pharmacodynamic study was performed with a single dose of 40 mg/kg IPI-926 to correlate IPI-926 plasma drug levels with pharmacodynamic changes in stromal Gli1 expression. As expected, the IPI-926 plasma levels correlated with the degree and duration of stromal Gli1 mRNA inhibition (Figure 5B). Importantly, stromal Gli1 mRNA did not exceed baseline levels as IPI-926 plasma levels dropped 24 hours after dosing (Figure 5B). Therefore, consistent with our in vitro findings, pathway hyperactivity was not observed with IPI-926 in vivo. Additionally, in a follow-up study, established MiaPaCa xenografts were treated with 40 mg/kg IPI-926 for 8 consecutive days after which IPI-926 administration was stopped; tumor growth rates did not exceed those of the vehicle controls when followed for an additional two weeks (data not shown).

## Discussion

The Hh pathway is dysregulated in a variety of human tumors and has prompted the development of Smo inhibitors to abrogate Hh signaling in a number of indications. This approach has proven to be successful in the treatment of locally advanced or metastatic basal cell carcinomas and Erivedge (vismodegib or GDC-0449), a Smo-antagonist, was recently approved for this indication. Recent studies have examined the effect of a variety of Smo antagonists on the subcellular localization of their target, Smo, after binding [14]. In contrast to vismodegib, Smo binding by the natural product cyclopamine, promotes Smo accumulation in the PC while maintaining its inhibitory effect on Hh signaling. It has been speculated that this mode of inhibition could have undesired clinical consequences if ciliary Smo accumulation leaves cells hyper-responsive to further Hh pathway activation after exposure of the inhibitor falls below a certain level (so-called “priming”) [14]. In tumor cells that are dependent upon Hh signaling for growth and survival, this hyper-responsiveness could then lead to enhanced tumor growth. Therefore, Smo inhibitors that prevent ciliary Smo accumulation, such as vismodegib might be preferable to Smo antagonists like cyclopamine that promote ciliary Smo accumulation. We present evidence that not all Smo antagonists that promote ciliary Smo accumulation show pathway hyper-responsiveness after compound withdrawal. Like cyclopamine, IPI-926 promoted ciliary Smo accumulation, however, in contrast to cyclopamine, IPI-926 pre-treatment did not lead to priming activity in any cell line tested. Additionally, cyclopamine-induced priming activity was detected only in the murine NIH-3T3 cell line, and priming was not observed with either cyclopamine or IPI-926 in the human HEPM cell line or in three additional Hh-responsive cell lines tested. Of these three additional cell lines, two were of murine origin and stromally-derived, similar to NIH-3T3 (C3H10T1/2 cells and a primary murine tumor stromal line), while the other was tumor derived from a human liposarcoma (SW-872). Therefore, while we could corroborate the cyclopamine-induced priming in murine NIH-3T3 cells reported by Wang et al [14], we did not observe this phenomenon in four other murine or human Hh-responsive cell lines tested under the same conditions. Importantly, in vivo human pancreatic xenograft studies performed with IPI-926 failed to show hyper-responsiveness of the Hh pathway or increases in tumor growth after compound withdrawal. Taken together, these data suggest that the priming phenomenon may be of limited relevance in a clinical setting.

**Figure 6:**
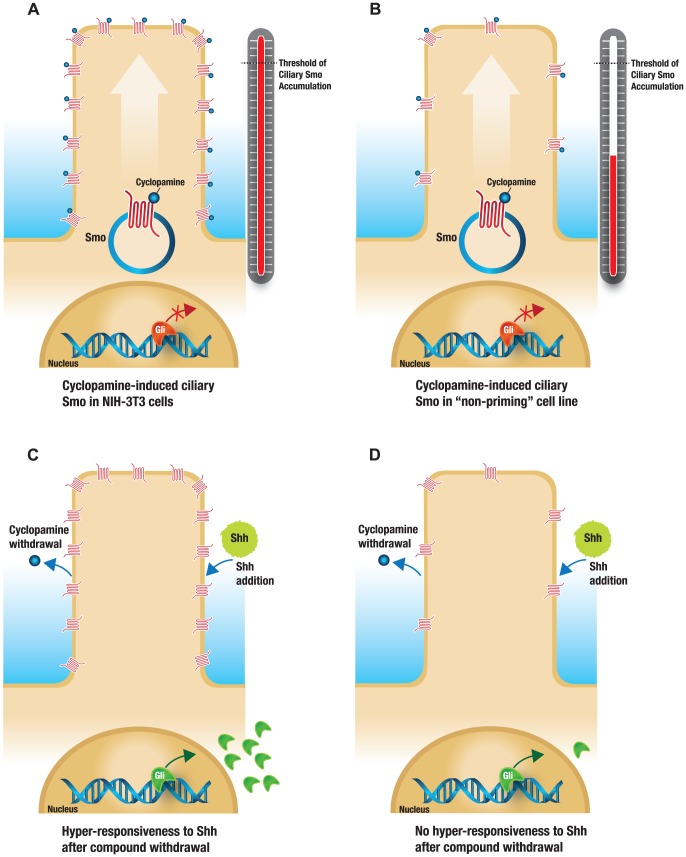
Model demonstrating threshold of Smo accumulation to PC necessary for priming effect. Cyclopamine induces ciliary Smo localization to a threshold necessary for priming activity to occur as observed in NIH-3T3 cells (A). In cell lines such as HEPM or C3H10T1/2, cyclopamine does not induce ciliary Smo localization to this threshold level (B). Upon cyclopamine withdrawal, followed by Shh addition, hyper-responsiveness as measured by Gli1 up-regulation is only observed in NIH-3T3 cells (C) and not cell lines where the required level of ciliary Smo accumulation was not achieved by cyclopamine pre-treatment (D).

Based on our comparison of the effects of cyclopamine and IPI-926 on Smo localization in the PC and the Hh pathway signaling after compound withdrawal in the NIH-3T3 and HEPM cell lines, we propose a model in which compound-induced ciliary Smo accumulation must reach a critical threshold in order for hyper-responsiveness to occur after compound removal (Figure 6). Only within NIH-3T3 cells, at supra-pharmacologic concentrations of cyclopamine (well above the EC_50_ in cell based assays [Figure 1]), was increased Smo observed in the PC compared to lower concentrations of cyclopamine (Figure 4A & C). The more potent inhibitor IPI-926 (at concentrations ranging from 0.01 nM to 10000 nM) was not capable of inducing the levels of ciliary Smo that correlated with cyclopamine-related priming activity in any of the cell lines tested (Figure 4B & D). Therefore, in NIH-3T3 cells, cyclopamine and IPI-926 treatment lead to different levels of ciliary Smo accumulation that directly correlated with cellular responsiveness to the priming effect. While cyclopamine inhibited Gli-reporter activity in the nanomolar range in both the human HEPM and murine NIH-3T3 cell lines, the priming activity only occurred in the murine cell line, raising the possibility that this phenomenon is only relevant in murine cells.

Recently, large-scale screening efforts have identified additional structurally diverse classes of Hh pathway inhibitors that distinctly antagonize the pathway. A subset of these Smo inhibitors disrupt PC formation, while others block Smo trafficking to the primary PC (like GDC-0449), and a third subset results in Smo accumulation in the primary PC (like cyclopamine and IPI-926) [16]. Additionally, certain glucocorticoids have also been shown to influence ciliary Smo localization and Hh pathway activity. Similar to the priming response seen with cyclopamine pre-treatment, NIH-3T3 cells pre-treated with 10 µM fluocinolone acetonide (FA), a concentration shown to promote ciliary accumulation of Smo but not activate the Hh pathway, followed by FA removal and Shh stimulation, showed a greater induction of Hh pathway activity compared to cells pre-treated with DMSO. The authors found that the EC_50_ for the FA induced priming response was approximately 4 µM, which was very close to the 5 µM EC_50_ required for efficient SMO accumulation in the PC [14]. These results are in agreement with our findings that a critical threshold of SMO accumulation much be reached in order for pathway hyper-responsiveness to occur.

With these new findings, further interrogation of the precise mechanism of action of these Hh pathway modulators is warranted to categorize them appropriately. Differential modes of pathway inhibition demonstrated by various classes of inhibitors may prove to be clinically useful in treating patients with acquired Smo resistance mutations such as SMO-D473H [17]. In addition, the structure of Smo bound to the Smo antagonist LY2940680 has been resolved [18]. This may allow a clearer determination of structure/function relationships among various Smo antagonists and their interaction with their target. In addition, Chen et al., has recently demonstrated that mammalian Smo is activated via multi-site phosphorylation of its carboxy-terminal tail which can influence its ciliary localization, conformation, and activity, [19] and antibodies have been developed against these phosphorylated or activated forms of Smo [19]. These tools will be important in further investigating the precise mechanism of action of Smo antagonists on Smo after the protein is activated. Investigation of activated Smo may also hold greater clinical relevance in the study of cancers with aberrant Hh activation, such as basal cell carcinoma.

## Materials and Methods

### Ethics Statement

This study was carried out in strict accordance with the recommendations in the Guide for the Care and Use of Laboratory Animals printed by the National Research Council of the National Academies. The protocol was approved by the Institutional Animal Care and Use Committee of Infinity Pharmaceuticals, Inc. Every effort was made to minimize animal suffering through the entirety of the study.

### Cell cultures

Unless otherwise noted, mammalian cell lines were obtained from the American Type Culture Collection (ATCC, Manassas, VA) and maintained in Dulbecco's Minimal Essential Medium (DMEM) (Life Technologies, Grand Island, NY) supplemented with 10% heat inactivated fetal bovine serum (FBS) (Sigma-Aldrich, St. Louis, MO) and 100 units/ml penicillin (Life Technologies, Grand Island, NY) and 100 µg/ml streptomycin (Life Technologies, Grand Island, NY) (complete DMEM). C3H10T1/2 cells were maintained in Basal Medium Eagle (BME) supplemented with 10% FBS, 100 units/ml penicillin and 100 µg/ml streptomycin plus 2 mM L-Glutamine (Life Technologies, Grand Island, NY). All cell cultures were maintained at 37°C in a humidified atmosphere of 5% CO_2_.

### Generation of Primary Murine Tumor Stroma

Primary murine tumor stroma was generated from L3.6pl pancreatic xenografts implanted subcutaneously in the flank of Ncr Nude male mice. Briefly, tumors were collected and manually homogenized into a single cell suspension. Cells were plated into 6-well plates at a density of 250,000 cells/ml in complete DMEM media. To selectively kill off the human L3.6pl tumor cells allowing for isolation of purified tumor stroma of murine origin, the cultures were treated with 50 ng/ml of diphtheria toxin (Sigma, St, Louis, MI) for 48–72 h [20]. The dead tumor cells were removed and the remaining murine tumor stroma were washed multiple times with complete DMEM before the cells were trypsinized and combined for continued passage.

### Generation of Shh Conditioned Medium

293 EcR Shh [JHU-64] cells were cultured in complete DMEM medium supplemented with 400 µg/ml G418 (Invitrogen, Carlsbad, CA) until reaching 80% confluence. The growth medium was then removed and DMEM containing 2% FBS was added back for 24 h, at which point the Shh conditioned medium (Shh CM) was collected and filtered through a 0.22 µm filter. Shh CM was stored at −80°C in small aliquots for future use.

### Gli-Luciferase Reporter Assay

NIH-3T3 cells were seeded into 6-well plates at 1.5×10^5^ cells/well. The following morning, cells were co-transfected with 8X Gli-Firefly Luciferase (Gift from Phil Beachey) or pRL-TK *Renilla* Luciferase control plasmids (Promega, Madison, WI) using GeneJuice transfection reagent (EMD Millipore, Billerica, MA). Six hours post transfection, cells were trypsinized and replated into 96-well plates. The following day, the media was replaced with low serum media containing 0.5% FBS. Cells were stimulated with 10% Shh conditioned media plus and minus compound as indicated. Luciferase activity was assayed 48 h later with the Dual-Glo luciferase assay system (Promega, Madison, WI). Values corresponding to the ratio of Firefly:Renilla luciferase activity were used to calculate percent inhibition relative to compound concentration and were plotted using GraphPad Prism software (GraphPad Software, Inc., La Jolla, CA).

The HEPM cells were stably transfected with the 8X Gli-Firefly Luciferase construct and were seeded directly into 96-well plates at 7500 cells/well. The following day, the media was replaced with low serum media containing 0.5% FBS and 24 h later compounds were added in low serum media with and without 20% Shh CM. Cells were assayed for firefly luciferase activity 48 h post stimulation as described above.

### Chemiluminescent Assay to Measure Alkaline Phosphatase Enzymatic Activity

C3H10T1/2 cells were seeded into 96-well plates at a density of 8×10^3^ cells/well and allowed to grow to confluence. Media containing 10% Shh CM with or without the addition of compound was then added and left on the cells for 72 h. To assay alkaline phosphatase production by the cells, 100 mL Tropix CDP-Star with Emerald II (Applied Biosystems, Grand Island, NY) was added per well and the plate was incubated at room temperature in the dark for 1 h. The plates were read on an EnVision plate reader at 405 nm. The percent inhibition with respect to compound concentration was plotted using GraphPad Prism software on a semi-log plot and EC_50_ values were determined by non-linear regression analysis with a four-parameter logistic equation.

### Smo Binding Assay

C3H10T1/2 mouse mesenchymal fibroblasts were transfected with a human Smo overexpression construct and assayed for binding 24 h later. Test compounds were added to the Smo-expressing cells with BODIPY-cyclopamine. Equilibrium was attained via a 90 minute preincubation. The cells were harvested and BODIPY fluorescence was evaluated by flow cytometry. Dose-response inhibition isotherms were generated utilizing the decrease in fluorescence with respect to increasing compound concentration. The data were scaled to maximum BODIPY-cyclopamine binding, determined by assessing BODIPY-cyclopamine staining of Smo-expressing cells in the absence of competitor compound.

### Gli1 Quantitative RT-PCR Priming Assay

NIH-3T3 or HEPM cells were seeded in 6-well format at 275,000 cells per well. The next day, cells were treated with various doses of cyclopamine or IPI-926 for 24 h followed by extensive washing with DMEM containing 0.5% FBS (low serum media). Wells were then re-fed with either low serum media or low serum media containing 100 nM rMShh (R&D Systems, Minneapolis, MN). RNA was harvested 21 h later using Qiagen RNeasy kit following the manufacturer's suggested protocol (Qiagen, Valencia, CA). Quantitative RT-PCR was performed using standard methods with an RNA to CT one-step kit (Qiagen, Valencia, CA). Inventoried gene expression assays for mouse and human GAPDH and Gli1 were ordered from Applied Biosystems (Grand Island, NY). Relative Gli1 levels were evaluated and normalized to GAPDH.

### Smoothened Ciliary Localization by Immunofluorescence

HEPM and NIH-3T3 cells were grown to confluence on glass coverslips and treated with 10% Shh conditioned media or compounds in DMEM supplemented with 0.5% FBS for 24 h. Cells were washed with PBS, fixed in cold 4% paraformaldehyde (Sigma, St, Louis, MI) for 20 minutes at room temperature, and permeabilized in 0.1% Triton X-100/PBS for 10 minutes. After rinsing with PBS, blocking was performed in 2.5% bovine serum albumin/PBS for 1 h at room temperature. Cells were stained with mouse anti-Smo (Clone E-5, 1∶50, Santa Cruz Biotechnology, Dallas, TX) and rabbit anti-acetylated α-tubulin (1∶250, Cell Signaling Technology, Danvers, MA) in 1% BSA/PBS for 2 h at room temperature (HEPM) or overnight at 4°C (NIH-3T3). Fixed cells were washed three times with 0.05% Tween 20/TBS and probed with anti-mouse Alexa Fluor 555 (1∶200, Invitrogen, Carlsbad, CA) and anti-rabbit Alexa Fluor 488 (1∶20, Invitrogen, Carlsbad, CA) in 1% BSA/PBS. Cells were washed as before and mounted on glass slides with DAPI ProLong Gold (Invitrogen, Carlsbad, CA). All immunofluorescent images were taken using a 100X (oil) objective using the TissueGnostics scanner (TissueGnostics, Tarzana, CA) at constant exposure times.

Specificity of the E-5 Smo antibody was confirmed by western blot analysis of 293 cells overexpressing either human or mouse full-length Myc-tagged Smo protein compared to untransfected 293 cells. Expected band sizes of approximately 85 kDa were confirmed using an anti-Myc antibody (data not shown).

### Quantification of Smo positive PC in NIH-3T3 and HEPM cells

The mean fluorescence intensity of ciliary Smo accumulation was calculated using Tissue Studio (Definiens, Carlsbad, CA) by adapting a method previously described [6]. Several different fields of view (FOVs) from slides of fluorescently stained cells were captured on the TissueGnostics (Tarzana, CA) and saved as jpeg image files (composite RGB, one stain per color). Analysis of the images was done in two passes. In the first pass, the cell nuclei stained with DAPI in the blue channel were identified and counted using “Nuclei Detection”. In the second pass, PC stained for acetylated tubulin were identified as if they were “nuclei” in green channel using “Nuclei Detection”. A neighborhood around each cilium was defined for measuring the red background signal as if it were “cytoplasm” using “Cell Simulation”. This allowed us to quantify the fluorescent signal in the red channel, representing Smo, where it co-localized with the green fluorescence from the cilium. The results from the two passes were merged using Spotfire (TIBCO Software, Somerville, MA), and the background corrected Smo signal and % ciliated cells calculated. Multiple fields of view were analyzed to obtain measurements on at least 90 or more cells per condition. 10 µM cyclopamine yielded the maximal Smo fluorescence intensity (63%) and all other data points were normalized to this max value. Finally, a dose-response curve was fit to the median values obtained for the different conditions.

### MiaPaCa subcutaneous xenograft model

MiaPaCa cells (10^7^ cells per mouse in 0.1 ml of sterile PBS) were implanted subcutaneously in the right flank of 5–6 week old Ncr Nude male mice (Taconic, Hudson, NY). Mice were housed 4 per cage and offered food and water ad libitum. Environmental controls for the animal room were set to maintain 18 to 26°C, a relative humidity of 30 to 70%, a minimum of 10 room air changes/hour, and a 12-hour light/12-hour dark cycle.

To determine the effect IPI-926 had on MiaPaCa tumor growth in vivo, mice were randomized into 2 treatment groups (N = 12 per group) when tumors reached an average volume of 200 mm^3^ in size. IPI-926 (40 mg/kg) formulated in 0.5% (v/v) hydroxypropyl methyl cellulose (HPMC) in sterile water or vehicle alone were orally administered once daily. Body weights and tumor caliper measurements (length and width) were recorded twice weekly. Tumor volume was calculated: tumor volume  =  (length x width^2^)/2.

### Analysis of murine Gli1 expression in the MiaPaCa Tumors

Mice were randomized (n = 3 mice per treatment group per time point) when tumors reached an average size of 300 mm^3^. Tumors were collected for pharmacokinetic and pharmacodynamic analysis at 0, 1, 2, 4, 8, 16, 24, 38, 48, 72 and 96 h after a single dose of 40 mg/kg IPI-926. For RNA isolation, frozen tumors were homogenized into a fine powder using the Geno/Grinder (SPEX, Metuchen, NJ) and total RNA was extracted using TRIzol (Invitrogen, Carlsbad, CA) followed by the RNeasy Midi Kit (Qiagen, Valencia, CA) and converted to cDNA using the High-Capacity cDNA Archive Kit (Applied Biosystems, Grand Island, NY). Quantitative RT-PCR was done using primers for murine GLI1 (Applied Biosystems, Grand Island, NY) on the 7900HT TaqMan real-time PCR instrument (Applied Biosystems, Grand Island, NY). Data were normalized to GAPDH.

### Plasma Sample Preparation and Quantitation of IPI-926 by LC-MS/MS analysis

All blood samples were collected into tubes containing sodium heparin, processed to plasma by centrifugation, and then frozen at −80°C. Prior to analysis, plasma samples were thawed on ice, 75 µL was transferred to a 96-well plate and two volumes of acetonitrile containing internal standard (deuterated IPI-926) were added. The samples were then placed on a plate shaker for 5 minutes, transferred to a MultiScreen Solvinert filter plate (Millipore, Milford, MA) and filtered into a 96-well plate using centrifugation. The filtrate was then diluted with an equal volume of water and transferred to a 96-well plate for liquid chromatography-mass spectrometry (LC-MS/MS) analysis. IPI-926 concentrations in the samples were determined from a calibration curve generated in blank plasma matrix. For quantitative analysis, peak area ratio of IPI-926 to internal standard was calculated and plotted against concentration.

The LC-MS/MS system used for sample analysis consisted of a CTC autosampler (Leap Technologies, Inc., Pittsboro, NC), Agilent 1100 LC pump (Agilent Technologies, Santa Clara, CA) and API-4000 mass spectrometer (Applied Biosystems, Foster City, CA). Sample injection volume was 20 µL on a C18 analytical column (Symmetry-shield IS C18, 2.1×20 mm, 3.5 µm, Waters Corp, Milford, MA). Bioanalytical samples were eluted using a 4 minute gradient from 5-95% acetonitrile in water, 0.1% formic acid. Multiple reaction monitoring (MRM) in positive ESI (Electrospray Ionization) mode was used to detect IPI-926 using the *m/z* transition of 505.4/114.1. The following MS parameters were used for sample analyses: source operating voltage was 5000 volts, source temperature was 500°C, nebulizer gas was 60 psi (nitrogen), de-clustering potential was 136 volts and collision energy was 55 volts. All data were acquired and processed using the Analyst 1.4.1 software (Applied Biosystems, Grand Island, NY).
